# Kikuchi-Fujimoto Disease Associated With Systemic Lupus Erythematosus Presenting With Prominent Cutaneous Manifestations: A Case Report

**DOI:** 10.7759/cureus.107099

**Published:** 2026-04-15

**Authors:** Imane Hakim, Layla Bendaoud, Maryem Aboudourib, Said Amal, Ouafa Hocar

**Affiliations:** 1 Department of Dermatology, Mohammed VI University Hospital, Biosciences Laboratory, Faculty of Medicine and Pharmacy, Marrakech, MAR

**Keywords:** cervical lymphadenopathy, cutaneous lupus erythematosus, kikuchi-fujimoto disease, necrotizing lymphadenitis, systemic lupus erythematosus

## Abstract

Kikuchi-Fujimoto disease is a rare necrotizing histiocytic lymphadenitis that typically affects young women and presents with febrile cervical lymphadenopathy. Because of its clinical and histopathological overlap with lupus adenitis and lymphoma, diagnosis may be challenging. We report the case of a 14-year-old girl admitted for prolonged fever and painful cervical lymphadenopathy associated with striking mucocutaneous manifestations. Dermatologic examination revealed massive bilateral periorbital edema leading to near-complete eyelid closure, diffuse centrofacial erythema forming infiltrated plaques, a morbilliform maculopapular eruption, malar butterfly erythema sparing the nasolabial folds, and painful erosive crusted cheilitis. Laboratory investigations showed lymphopenia, anemia, thrombocytopenia, hyperferritinemia, and elevated lactate dehydrogenase levels. Autoimmune workup revealed positive antinuclear antibodies and anti-double-stranded DNA antibodies with low complement levels. Imaging demonstrated cervical and axillary polyadenopathy associated with moderate ascites. Lymph node biopsy confirmed Kikuchi-Fujimoto disease, while skin biopsy supported the diagnosis of systemic lupus erythematosus with cutaneous manifestations. The clinical course was complicated by encephalopathy requiring treatment with high-dose corticosteroids, intravenous immunoglobulins, hydroxychloroquine, and etoposide, leading to progressive improvement. This case highlights a possible association between Kikuchi-Fujimoto disease and systemic lupus erythematosus with cutaneous manifestations. It emphasizes the key diagnostic value of dermatologic examination in identifying associated connective tissue disease and guiding early multidisciplinary management.

## Introduction

Kikuchi-Fujimoto disease, also known as necrotizing histiocytic lymphadenitis, is a rare benign condition first described in Japan in 1972. It predominantly affects young women and typically presents with fever and cervical lymphadenopathy. The disease is generally self-limited, although atypical and severe presentations may occur. Although the exact etiology remains unclear, both infectious and autoimmune mechanisms have been proposed [1,2].

Kikuchi-Fujimoto disease may clinically mimic serious conditions such as lymphoma, tuberculosis, or autoimmune diseases, particularly systemic lupus erythematosus, making the diagnosis challenging [3]. Increasing evidence suggests a possible relationship between Kikuchi-Fujimoto disease and lupus erythematosus, as both conditions share several clinical and histopathological features [4].

Several reports have described different patterns of association between Kikuchi-Fujimoto disease and lupus erythematosus, including cases where lupus erythematosus precedes Kikuchi-Fujimoto disease, occurs simultaneously, or develops later during follow-up [5]. Dermatological manifestations may therefore represent important diagnostic clues suggesting an underlying autoimmune disorder [6].

We report a rare and severe pediatric case of Kikuchi-Fujimoto disease associated with systemic lupus erythematosus, highlighting the diagnostic challenges and the importance of dermatological examination in guiding early multidisciplinary management.

## Case presentation

A 14-year-old girl was admitted for a prolonged fever evolving over three months. The initial symptoms began with the appearance of a painful left cervical lymphadenopathy associated with febrile headaches. She initially received antibiotic therapy and corticosteroids for suspected inflammatory or autoimmune disease, without clinical improvement. During a previous hospitalization, a lumbar puncture revealed aseptic meningitis with 40 cells showing a mixed formula, for which meningitis-dose antibiotics and corticosteroids were administered.

**Figure 1:**
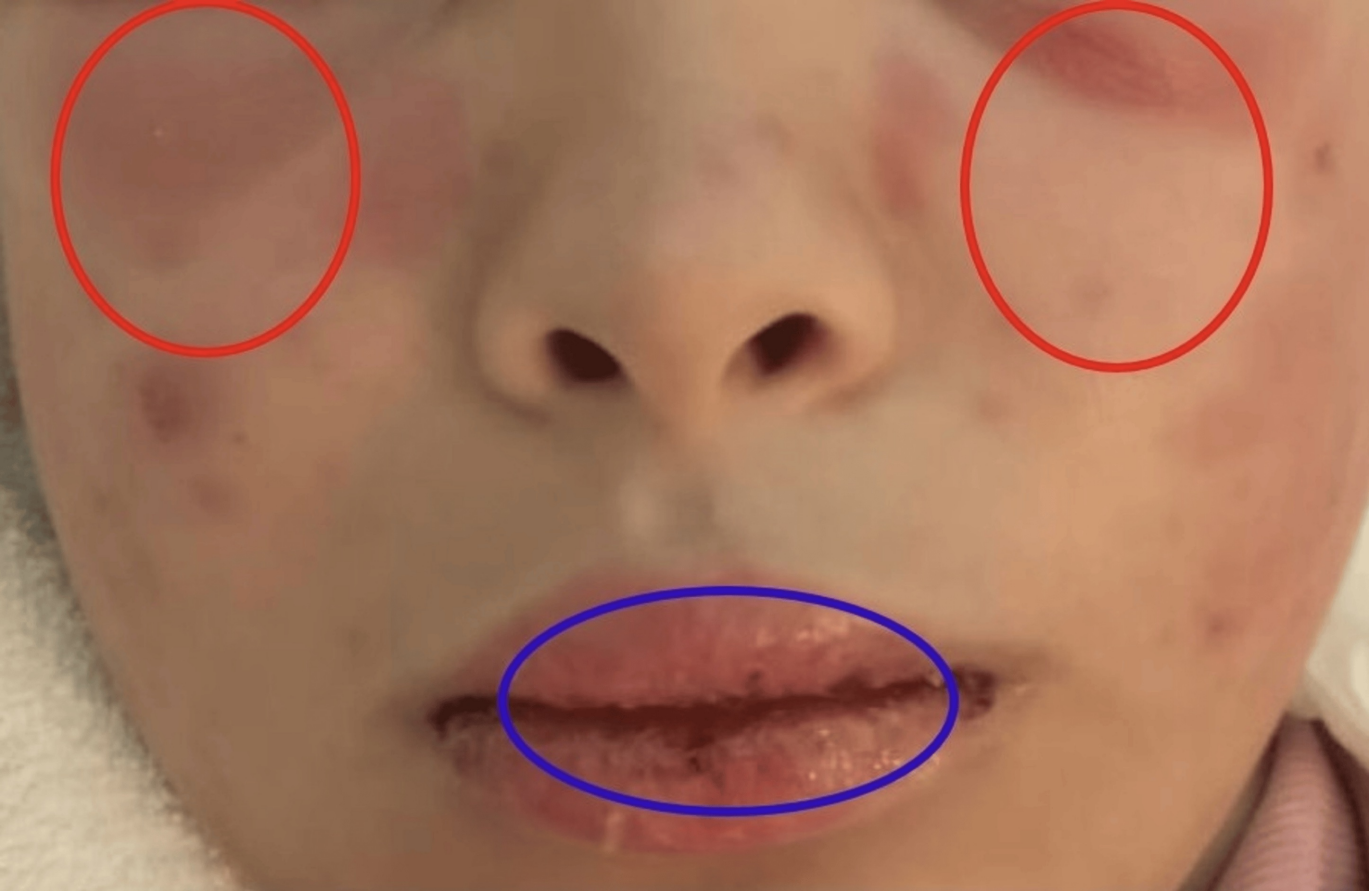
Severe bilateral periorbital edema (red circles) associated with centrofacial erythema and erosive cheilitis (blue circle) in a 14-year-old patient with Kikuchi-Fujimoto disease associated with systemic lupus erythematosus, illustrating prominent mucocutaneous involvement

Upon admission to our department, the patient presented with a persistent fever reaching 39°C associated with headaches and marked asthenia. Dermatological examination revealed erosive cheilitis and stomatitis, centrofacial erythema involving the malar and frontal regions, a morbilliform eruption affecting the arms and thorax, bilateral periorbital erythematous edema without conjunctival injection, and periungual erythema. Hemodynamic and respiratory status remained stable (Figure 1).

Laboratory investigations revealed lymphopenia, microcytic hypochromic anemia (hemoglobin 7 g/dL), thrombocytopenia (86,000/mm³), elevated lactate dehydrogenase (850 U/L), hyperferritinemia (>1600 ng/mL), and elevated C-reactive protein (12 mg/L at admission, later increasing to 52.3 mg/L). Procalcitonin was negative. Electrolyte levels and renal and hepatic function tests were within normal limits. Autoimmune workup revealed positive antinuclear antibodies (1:640), positive anti-double-stranded DNA antibodies (85 IU/mL), and low complement levels (C3: 0.55 g/L, C4: 0.08 g/L). Urinalysis showed proteinuria of 280 mg/L, with a protein-to-creatinine ratio of 27 mg/mmol. Given the suspicion of macrophage activation syndrome, triglyceride levels were elevated (3.2 mmol/L), and fibrinogen levels were decreased (1.2 g/L). Bone marrow examination revealed hemophagocytosis, supporting the diagnosis of macrophage activation syndrome. Laboratory findings are summarized in Table 1.

**Table 1:** Laboratory findings ANA: antinuclear antibody, anti-dsDNA: anti-double-stranded deoxyribonucleic acid antibody, C3: complement component 3, C4: complement component 4, MAS: macrophage activation syndrome

Parameter	Result	Reference range	Interpretation
Hemoglobin	7 g/dL	12-16 g/dL	Anemia
White blood cells	Lymphopenia	-	Cytopenia
Platelets	86,000/mm³	150,000-400,000/mm³	Thrombocytopenia
Lactate dehydrogenase	850 U/L	<250 U/L	Elevated
Ferritin	>1600 ng/mL	15-150 ng/mL	Hyperferritinemia
C-reactive protein	12-52.3 mg/L	<5 mg/L	Elevated
Procalcitonin	Negative	-	No bacterial infection
ANA	Positive (1:640)	Negative	Autoimmune marker
Anti-dsDNA	Positive (85 IU/mL)	<30 IU/mL	Lupus activity
Complement C3	0.55 g/L	0.9-1.8 g/L	Low
Complement C4	0.08 g/L	0.1-0.4 g/L	Low
Proteinuria	280 mg/L	<150 mg/L	Renal involvement
Protein/creatinine ratio	27 mg/mmol	<15 mg/mmol	Elevated
Triglycerides	3.2 mmol/L	<1.7 mmol/L	MAS compatible
Fibrinogen	1.2 g/L	2-4 g/L	MAS compatible
Bone marrow	Hemophagocytosis	-	MAS compatible

Radiological investigations, including thoraco-abdomino-pelvic computed tomography, demonstrated cervical and axillary lymphadenopathy associated with moderate ascites. Brain magnetic resonance imaging showed findings compatible with post-encephalitic changes, with no radiological evidence of optic nerve or orbital involvement. The absence of optic nerve or orbital abnormalities on imaging suggested that the visual impairment was more likely related to central nervous system involvement rather than primary optic neuropathy.

Excisional biopsy of a left cervical lymph node excluded malignant lymphoma and confirmed necrotizing histiocytic lymphadenitis consistent with Kikuchi-Fujimoto disease. In contrast, a skin biopsy supported the diagnosis of systemic lupus erythematosus with cutaneous manifestations, consistent with systemic involvement. Infectious causes were investigated, including viral serologies for Epstein-Barr virus, cytomegalovirus, human immunodeficiency virus, and parvovirus B19, as well as bacterial investigations. All results were negative.

During hospitalization, the patient’s condition progressively deteriorated with the onset of altered consciousness and marked somnolence, with the Glasgow Coma Scale score decreasing to 10/15. She was subsequently transferred to the pediatric intensive care unit. High-dose corticosteroid therapy with intravenous methylprednisolone pulses was initiated first due to suspected inflammatory and autoimmune disease. As the clinical condition deteriorated and macrophage activation syndrome was suspected, intravenous immunoglobulins were subsequently administered. Due to persistent neurological involvement and worsening laboratory parameters, etoposide was introduced as rescue therapy. Hydroxychloroquine was initiated following stabilization. Electroencephalography demonstrated diffuse cerebral slowing consistent with inflammatory encephalopathy. The encephalopathy was considered multifactorial, with contributions from systemic lupus erythematosus and macrophage activation syndrome, given the presence of neurological involvement, cytopenias, hyperferritinemia, and autoimmune markers.

Following initiation of immunosuppressive therapy, gradual clinical stabilization and progressive improvement of the cutaneous manifestations were observed.

## Discussion

Kikuchi-Fujimoto disease is a rare benign condition characterized by necrotizing histiocytic lymphadenitis that predominantly affects young women [1]. The disease usually presents with fever and cervical lymphadenopathy and may clinically mimic infections, lymphoma, or autoimmune diseases, making the diagnosis particularly challenging [3].

Large case series have described the clinical and pathological characteristics of this condition and highlighted the importance of lymph node biopsy in establishing the diagnosis [2,4].

The association between Kikuchi-Fujimoto disease and systemic lupus erythematosus has been increasingly reported in the literature. Several authors have suggested a possible association between Kikuchi-Fujimoto disease and autoimmune dysregulation in certain patients [5].

Three main clinical scenarios have been described: lupus erythematosus may precede Kikuchi-Fujimoto disease, occur simultaneously, or develop after the episode of necrotizing lymphadenitis [5]. Cutaneous manifestations associated with Kikuchi-Fujimoto disease have also been reported and may mimic autoimmune dermatological disorders [6,7].

In our patient, lymph node biopsy demonstrated necrotizing lymphadenitis consistent with Kikuchi-Fujimoto disease without evidence of malignancy. However, the presence of marked mucocutaneous manifestations, including malar erythema, morbilliform rash, and erosive cheilitis, strongly suggested an associated connective tissue disease. Skin biopsy and direct immunofluorescence supported the diagnosis of systemic lupus erythematosus.

Additionally, the presence of cervical and axillary lymphadenopathy associated with systemic manifestations was consistent with a generalized form of Kikuchi-Fujimoto disease. This distinction is clinically important, as generalized disease may be associated with a more severe clinical course and may require systemic treatment rather than surgical management alone.

Neurological involvement in Kikuchi-Fujimoto disease is uncommon but has been described in association with aseptic meningitis and encephalopathy [8,9]. In our patient, cerebrospinal fluid analysis revealed aseptic meningitis, while brain magnetic resonance imaging demonstrated meningeal enhancement, and electroencephalography showed diffuse cerebral slowing suggestive of inflammatory central nervous system involvement. These findings, together with systemic features, were more consistent with systemic lupus erythematosus rather than isolated cutaneous lupus erythematosus.

Management of isolated Kikuchi-Fujimoto disease is usually supportive because the condition is often self-limited. However, when associated with systemic autoimmune manifestations, immunosuppressive therapy may be required [10]. Recent reviews have also emphasized the importance of early recognition and multidisciplinary management of this condition [11].

This case highlights the importance of careful dermatological examination in patients presenting with Kikuchi-Fujimoto disease, as mucocutaneous manifestations may be a key clue to an associated autoimmune disorder, such as systemic lupus erythematosus with cutaneous manifestations.

This report has several limitations. The temporal association between Kikuchi-Fujimoto disease and systemic lupus erythematosus does not establish causality. Alternative diagnoses, including isolated systemic lupus erythematosus with lymphadenopathy and macrophage activation syndrome, were considered. However, histopathological findings supported the coexistence of Kikuchi-Fujimoto disease and lupus erythematosus. Further studies are needed to better understand the relationship between these conditions.

## Conclusions

This case highlights a possible association between Kikuchi-Fujimoto disease and systemic lupus erythematosus in an adolescent with an unusually severe clinical presentation. It underscores the importance of careful clinical evaluation, particularly in pediatric patients presenting with systemic symptoms and atypical mucocutaneous findings. Early recognition and multidisciplinary management are essential to optimize outcomes. However, given the nature of a single case report, this observation should be interpreted with caution, and further studies are needed to better understand the relationship between these conditions.

## References

[1] Bosch X, Guilabert A (2006). Kikuchi-Fujimoto disease. Orphanet J Rare Dis.

[2] Kucukardali Y, Solmazgul E, Kunter E, Oncul O, Yildirim S, Kaplan M (2007). Kikuchi-Fujimoto disease: analysis of 244 cases. Clin Rheumatol.

[3] Perry AM, Choi SM (2018). Kikuchi-Fujimoto disease: a review. Arch Pathol Lab Med.

[4] Hutchinson CB, Wang E (2010). Kikuchi-Fujimoto disease. Arch Pathol Lab Med.

[5] Baenas DF, Diehl FA, Haye Salinas MJ, Riva V, Diller A, Lemos PA (2016). Kikuchi-Fujimoto disease and systemic lupus erythematosus. Int Med Case Rep J.

[6] Rezai K, Kuchipudi S, Chundi V, Ariga R, Loew J, Sha BE (2004). Kikuchi-Fujimoto disease: hydroxychloroquine as a treatment. Clin Infect Dis.

[7] Mahajan VK, Sharma V, Sharma N, Rani R (2023). Kikuchi-Fujimoto disease: a comprehensive review. World J Clin Cases.

[8] Yen HR, Lin PY, Chuang WY, Chang ML, Chiu CH (2004). Skin manifestations of Kikuchi-Fujimoto disease: case report and review. Eur J Pediatr.

[9] Liu B, Sun Y, Hu B, Shi WY, Chen TM, Liu LL, Liu G (2025). Kikuchi-Fujimoto disease concurrent with aseptic meningitis or encephalitis in children: a case-control study. BMC Pediatr.

[10] Deaver D, Horna P, Cualing H, Sokol L (2014). Pathogenesis, diagnosis, and management of Kikuchi-Fujimoto disease. Cancer Control.

[11] Motwani J, Kumar A, Azhar L (2025). Diagnostic challenges and management of kikuchi-fujimoto disease: a rare case report. Ann Med Surg (Lond).

